# Effects of exosomes derived from *Trichinella spiralis* infective larvae on intestinal epithelial barrier function

**DOI:** 10.1186/s13567-022-01108-y

**Published:** 2022-10-22

**Authors:** Ruibiao Wang, Yuheng Zhang, Jingbo Zhen, Jinpeng Zhang, Zixuan Pang, Xuewei Song, Lihao Lin, Feng Sun, Yixin Lu

**Affiliations:** Heilongjiang Provincial Key Laboratory of Zoonosis, College of Veterinary Medicine, Northeast Agricultural University, 600 Changjiang Street, Harbin, 150030 China

**Keywords:** Exosomes, *Trichinella spiralis*, barrier function, tight junction

## Abstract

Muscle larvae of *Trichinella spiralis* parasitize the host intestinal epithelium. The mechanisms of exosomes participating in the invasion of *T. spiralis* muscle larvae are unclear. Hence, the purpose of this study was to explore the effect of exosomes derived from *T. spiralis* infective larvae (*Ts*Exos) on the barrier function of porcine small intestinal epithelial cells (IPEC-J2). First, *Ts*Exos were successfully obtained, and their ingestion by epithelial cells was validated. Furthermore, the optimal induction condition was determined by the CCK8 kit, and we found that exposure to 150 μg/mL *Ts*Exos for 12/24 h decreased the viability of IPEC-J2 cells by 30%. Based on this outcome, the effects of *Ts*Exos on cell biological processes and tight junctions were studied. After coincubation of *Ts*Exos and IPEC-J2 cells, the results showed a significant increase in the content of FITC-dextran and in the levels of lactate dehydrogenase (LDH) and reactive oxygen species (ROS). The rate of apoptosis increased by 12.57%, and nuclear pyknosis and nuclear rupture were observed. After the cells were induced by *Ts*Exos, the expression of IL-1 was upregulated, but the expression of IL-10, TGF-β, TLR-5, MUC-1 and MUC-2 was downregulated. *Ts*Exo induction also led to a decrease in the levels of ZO-1, CLDN-3, and OCLN. In conclusion, *Ts*Exos are involved in several cellular biological processes, and they function by disrupting physiological and biochemical processes, hyperactivating innate immunity, and damaging tight junctions.

## Introduction

*Trichinella spiralis* is a zoonotic parasitic disease found worldwide [1]. In the intestinal infection stage of *T. spiralis*, it invades multiple cells by sensing appropriate ligands [2]. The process of invasion may subsequently lead to cell membrane rupture and cytoplasmic destruction, thereby inducing apoptosis and causing changes in membrane permeability [3, 4]. Additionally, to resist the invasion of *T. spiralis*, the host activates the autoimmune system; however, its overreaction can be harmful to the host itself [3].

Recent studies have reported that most parasites can secrete exosomes that can deliver their cargo to host cells upon ingestion [5–9]. Exosomes derived from parasites, when transferred to host cells, can create a conducive microenvironment for parasite immune escape by regulating host cell proliferation, cell signalling pathways, and gene expression [10, 11].

Song et al. found that serine proteases and cysteine proteases in the excretory/secretory proteins (ESPs) of *T. spiralis* destroyed gut epithelial integrity by degrading tight junction proteins and played key roles in *T. spiralis* invasion, growth and survival in the host [12]. As an important component of ESPs, exosomes are involved in the regulation of many biological functions; however, their roles in modulating the functions of host intestinal epithelial cells in *T. spiralis* infection are unclear. Therefore, the purpose of this study was to explore the effect of exosomes derived from *T. spiralis* muscle larva (*Ts*Exos) on the barrier function of intestinal epithelial cells during the invasion of *T. spiralis* to facilitate its parasitic process. The results of this study will provide useful insights for exploring the invasion process.

## Materials and methods

### Animals, parasites, and cell culture

Six- to eight-week-old female KM mice were purchased from Harbin Medical University. All animal husbandry and experimental procedures were performed in accordance with the Chinese Animal Management Ordinance, and the animal experiment standards were approved by the Animal Management Committee of Northeast Agricultural University. All mice had free access to food and water, and they were maintained under SPF conditions with humidity of 70 ± 10% and temperature of 20 ± 2 ℃.

*Trichinella spiralis* (strain ISS533) was cultured in KM mice, and muscle larvae (ML) were isolated from the muscles of infected KM mice by the standard method as described previously [13].

Porcine small intestinal epithelial cells (IPEC-J2) were donated by Harbin Veterinary Research Institute and grown using 90% DMEM (Meilunbio, China) with 10% foetal bovine serum (FBS, PAN, Germany) and 1% 100 U/mL penicillin/streptomycin (Solarbio, China).

### Isolation and identification of *Ts*Exos

*Trichinella spiralis* ML were collected from mouse muscle on the 40^th^ day post-infection and then cultured in RPMI-1640 medium (Meilunbio, China) with 1% 100 U/mL penicillin/streptomycin at 37 ℃ and 5% CO_2_ for 48 h. The exosomes secreted in the culture supernatant were collected by the ultracentrifugation method as described by Wei et al. [14]. The concentration of *Ts*Exos was measured by a BCA protein assay kit (Meilunbio, China). The shape of the exosomes was observed by a transmission electron microscope (TEM) (Hitachi, Japan). The size of *Ts*Exos was measured using a nanoparticle tracking analyser (NTA) (NanoFCM). The specific marker CD63 of *Ts*Exos was determined by Western blots.

### Uptake of *Ts*Exos by IPEC-J2 cells

To verify the internalization of *Ts*Exos by IPEC-J2 cells, we seeded cells in 24-well plates (approximately 1 × 10^5^ cells per well) and cultured them with advanced DMEM at 37 ℃ and 5% CO_2_ for 12 h. The *Ts*Exos (300 μg/mL) and PBS (control) were labelled using a PKH26 kit (Aidisheng, China) as described by Liu et al. [15], in which the exosomes were labelled red. The labelled *Ts*Exos were incubated with IPEC-J2 cells for 6, 12, 18, and 24 h. Thereafter, the cells were fixed with 4% paraformaldehyde (Biosharp, China), washed with PBS, permeabilized with 0.25% Triton X-100 (Biofroxx, Germany), and stained with 10% DAPI (Solarbio, China). The cells were examined by a fluorescence microscope, and images were captured (Syngene, USA).

### Determination of the optimal reaction concentration and uptake time between *Ts*Exos and IPEC-J2 cells

A CCK-8 kit (Meilunbio, China) was used to detect cell viability as described earlier [16]. Briefly, cells were seeded in 96-well plates (approximately 1 × 10^3^ cells per well) and cultured under the same conditions as above. *Ts*Exos (20, 50, 100, 150, 200, 300 μg/mL) or PBS was cocultured with IPEC-J2 cells for 3, 6, 12, 24, 48 h. Then, 10 μL of CCK-8 solution was added to each well, and the cell culture plate was incubated for 2 h. The absorbance at 450 nm was measured using a plate reader (BioTek, USA).

### Measurement of permeability

The permeability of the IPEC-J2 cell monolayer was measured by performing a FITC-dextran assay (40 kDa, Yuanyebio, China) [17]. Cells were seeded in a Transwell chamber with 0.4 μm pores (Labselect, China) that had been placed in a 6-well plate at a density of 5 × 10^5^ cells/well. Cells were cultured in the medium until completely differentiated. Then, *Ts*Exos (150 μg/mL) were added to the cells for 3, 6, 12, 18, and 24 h. FITC-dextran (5 mg/mL) was added to the apical compartment of the Transwell insert for 5 h. Subsequently, the basolateral medium (no FBS) was collected into black CellCarrier-96 microplates (PerkinElmer, USA) for the measurement of fluorescence at 493-nm excitation and 517-nm emission wavelengths using a microplate reader (Tecan, Switzerland).

### Quantitative real-time PCR

The relative expression of genes in IPECs was evaluated via quantitative real-time PCR (RT‒qPCR). The primer sequences for each detected gene are shown in Table 1. Cell preparation and treatments were the same as above. Total RNA was extracted from IPECs using a total RNA extraction kit (Solarbio, China). After synthesis of cDNA from total RNA using the Prime Script 1^st^ Strand cDNA Synthesis Kit (TaKaRa, Japan), RT-qPCRs were performed using the Roche Light Cycler 480 system. The results were calculated using the 2^−∆∆Ct^ method [18].

**Table 1:** **Primers of the detected genes**.

Gene name	Primers	Sequence	Access number
GAPDH	Forward	5′-GGTGAAGGTCGGAGTGAACG-3′	NM_001206359.1
	Reverse	5′-CCGTGGGTGGAATCATACTGG-3′	
IL-1β	Forward	5′-AAAGATAACACGCCCACCC-3′	NM_214055.1
	Reverse	5′-GGAGTTTCCCAGGAAGACG-3′	
TNF-α	Forward	5′-GCTCTTCTGCCTACTGCACTTC-3′	NM_214022.1
	Reverse	5′-GCTGTCCCTCGGCTTTGA-3′	
TGF-β	Forward	5′-ATTTACTTACTGAGCATCTTGGACCTTA-3′	NM_214015.2
	Reverse	5′-GGGTGTTATCAGAGTCCCTTTTAGC-3′	
IL-10	Forward	5′-GACTCAACGAAGAAGGCACAG-3′	NM_214041.1
	Reverse	5′-GCAGGCTGGTTGGGAAGT-3′	
ZO-1	Forward	5′-AGGTGCTCCCATCGT-3′	XM_021098827
	Reverse	5′-TTTCGGTAATACTCTTCATC-3′	
ZO-2	Forward	5′-GGAGCATTGACCCGACTTAC-3′	NM_001206404
	Reverse	5′-AGACCATACTCTTCATTCGCTTT-3′	
CLDN-3	Forward	5′-CAGAGCCGTTCGCAACCAGG-3′	NM_001160075.1
	Reverse	5′-CACCACGCAGTTCATCCACAGG-3′	
Occludin	Forward	5′-CACCCAGCAACGACA-3′	NM_001163647
	Reverse	5′-ATAACGAGCATAGACAGAAT-3′	
TLR1	Forward	5′-CTCTGCTCAAGGACTTCCGTGTA-3′	NM_001031775.1
	Reverse	5′-AGAGCCAGTGCCAGCCCAGT-3′	
TLR2	Forward	5′-GGTCCGATGCTGGTCTTTATC-3′	NM_213761.1
	Reverse	5′-GCAAGTCACCCTTTATGTTATTCA-3′	
TLR4	Forward	5′-CCAGTGCTGCTTTGAATAGAG-3′	NM_001293316.1
	Reverse	5′-GAACAGAAGTGACCCGGAGA-3′	
TLR5	Forward	5′-CCAACACCCTTTCTCCAGCAT-3′	NM_001348771.1
	Reverse	5′-GATAGGACGCACGCCTCTTT-3′	
MUC-1	Forward	5′-ATGAGCTGGGAGCACAGGTGG-3′	XM_001926883.5
	Reverse	5′-CCAGGCTCGGATGGACTTCG-3′	
MUC-2	Forward	5′-AGGACGACACCATCTACCTCACTCA-3′	XM_013989745.1
	Reverse	5′-GCAAGGCCAGCTCGGGAAT-3′	

### Western blotting

Total proteins were extracted from IPEC-J2 cells with RIPA lysis buffer and 1% PMSF (Solarbio, China) at a ratio of 150–250 μL of lysis buffer per well. The protein concentration was examined using a BCA assay kit. The proteins were boiled with 5 × SDS‒PAGE sample loading buffer (Biosharp, China) and separated using SDS‒PAGE. The proteins were blotted onto a polyvinylidene fluoride filter membrane (Millipore, USA). The membrane was placed in blocking buffer (5% nonfat milk) for 2 h at room temperature. Subsequently, the membrane was incubated with a solution of the appropriate primary antibodies (Wanleibio, China; Bioss, China; Abclonal, USA) overnight at 4 °C. After three washes with PBST, the membrane was incubated with the respective horseradish peroxidase (HRP)-conjugated secondary antibody (Abclonal, USA), which was diluted in 5% nonfat milk (1:5000) for 2 h at room temperature. Finally, the blot was developed using the ultrasensitive ECL chemiluminescence reagent (Meilunbio, China), and the membrane was exposed. The bands were quantified using a chemiluminescence imaging system (Syngene, USA) and analysed with ImageJ.

### Cell immunofluorescence assay

After induction with *Ts*Exos, the cells were washed three times with PBS, fixed with 4% paraformaldehyde for 30 min and then permeabilized with 0.25% Triton X-100 for 10 min. Blocking was performed with 2% bovine serum albumin for 30 min at room temperature. Thereafter, the cells were rinsed with PBS and incubated with primary antibodies against ZO-1 (1:300), CLDN-3 (1:300) and OCLN (1:300) overnight at 4 °C. Subsequently, the wells were rinsed three times with PBS and incubated with FITC-labelled secondary antibodies (1:300, Bioss, China) for 1 h at room temperature in the dark. Then, cell nuclei were stained with 2 μg/mL DAPI for 8 min in a dark environment. Finally, the cells were observed under a fluorescence microscope.

### Detection of cytotoxicity, oxidative stress, and apoptosis

Cytotoxicity induced by *Ts*Exos was assessed by lactate dehydrogenase (LDH) leakage into the culture medium using an LDH Assay kit (Beyotime, China). First, IPECs were cultured in plates with different wells for 12 h. Then, *Ts*Exos (150 μg/mL) or PBS was used to induce cells for 12 and 24 h. Then, the influence of *Ts*Exos on cytotoxicity was evaluated by an LDH assay kit according to the instructions. Additionally, according to the technical manual of a Reactive Oxygen Species Assay kit (Meilunbio, China), the levels of oxidative stress were detected by measuring fluorescence at 488-nm excitation and 525-nm emission wavelengths and by observing the fluorescence under a fluorescence microscope. Furthermore, apoptotic cells were detected using an Annexin V-FITC/PI cell apoptosis kit (Meilunbio, China) by FCM, in which the cells that were stained with PI and FITC were identified as apoptotic cells. Hoechst 33258 (Leagene, China) staining was performed to confirm apoptosis, and the intensity of blue fluorescence was observed [19]. Moreover, Western blotting was used to detect the expression of apoptotic genes (Bax, Bcl-2, purchased from Wanleibio, China) (refer to the method in the section “Western blotting”).

### Statistical analysis

All data are expressed as the mean ± SD. Statistical analysis was performed using GraphPad Prism 5. ImageJ software was used to quantify the band intensities. Differences between groups were assessed by one-way analysis of variance (ANOVA) in SPSS 22 software. *P* < 0.05 was considered statistically significant.

## Results

### Characterization of *Ts*Exos and uptake by IPECs

**Figure 1:**
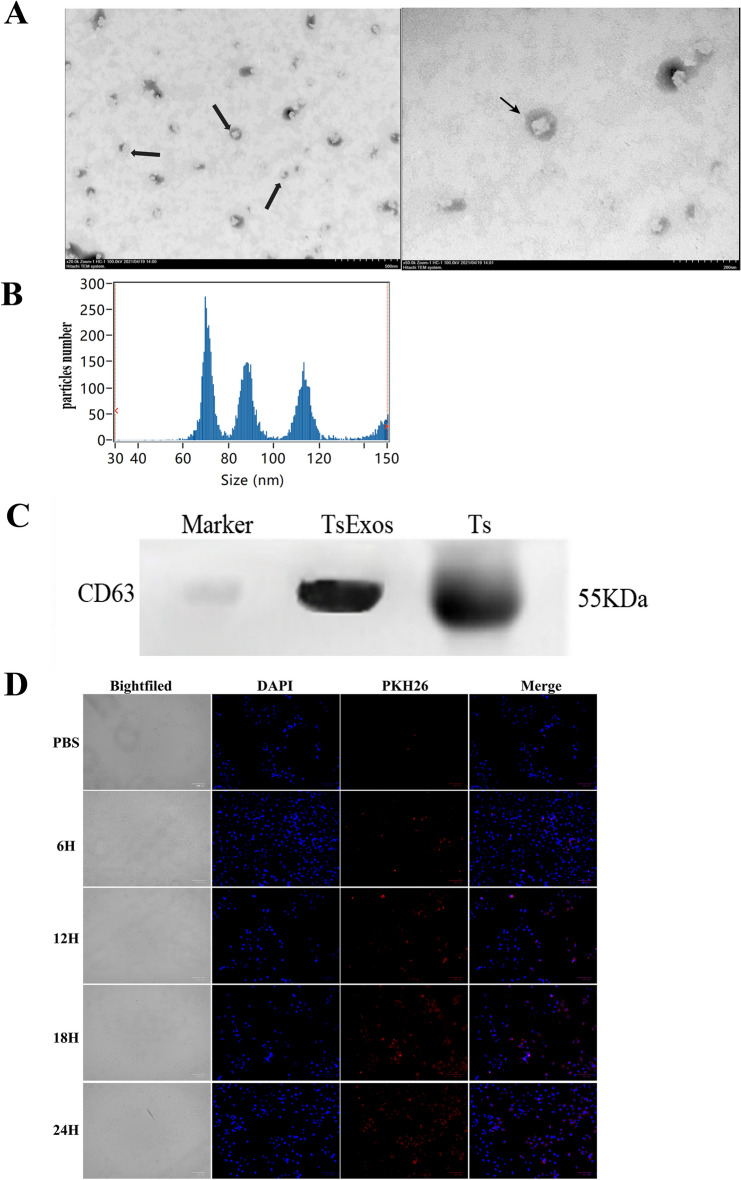
**Identification of TsExos and uptake by IPECs. A** TEM images of *Ts*Exos. The left figure shows a magnification of 20K× and a scale bar of 50 nm, and the right figure shows a magnification of 50K× and a scale bar of 20 nm. **B** Size detected by nanoparticle tracking analysis (NTA). **C** Identification of CD63 in Exos by Western blot analysis. **D** Uptake of *Ts*Exos by IPECs detected using fluorescence microscopy. DAPI staining of the nucleus was green, and the PKH26 dye was red.

To explore the effect of exosomes derived from *T. spiralis* muscle larvae (*Ts*Exos) on the intestinal epithelial barrier, we isolated exosomes from *T. spiralis* using a classical ultracentrifugation method. TEM and NTA showed round and typically cup-shaped exosomes, as reported earlier [20], with most membrane structure sizes ranging between 60 and 120 nm (Figures 1A and B). Western blot analysis demonstrated the existence of the specific marker protein CD63 (Figure 1C). Together, these results indicated the successful acquisition of *Ts*Exos. Then, to determine whether *Ts*Exos were taken up by IPECs, we incubated PKH26-labelled *Ts*Exos with IPEC-J2 cells. As shown in Figure 1D, in the physiological state, *Ts*Exos were significantly endocytosed by epithelial cells at 6 h, and the number of exosomes ingested showed a significant time-dependent increase, especially at 24 h, indicating that the exosomes could enter IPEC-J2 cells.

### *Ts*Exos affected the proliferation of IPECs

**Figure 2:**
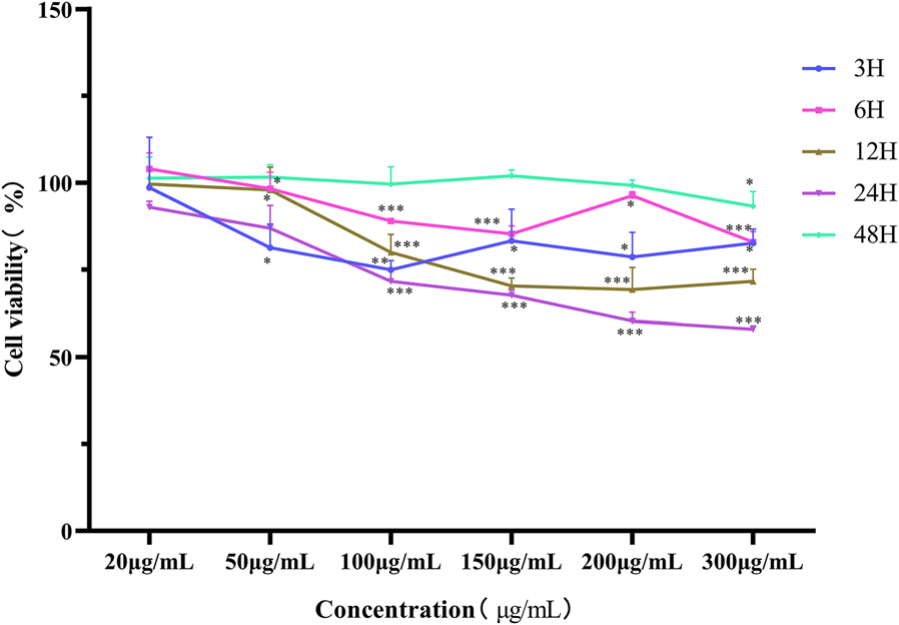
**Measurement of cell proliferation.** The *Ts*Exos (20, 50, 100, 150, 200, 300 μg/mL) were incubated with IPEC-J2 cells for 3, 6, 12, 24, 48 h to evaluate cell viability. The OD_450_ values were considered the cell proliferation index. The data were analysed from 3 independent experiments, and the data are expressed as the mean ± SD. *P < 0.05, **P < 0.01, ***P < 0.001 compared with the IPEC + PBS group.

The influence of *Ts*Exos on cell proliferation was evaluated by a CCK8 assay kit. The results highlighted that the cells exposed to 20 μg/mL *Ts*Exos showed no change in proliferation (*P* > 0.05). However, the cell viability was significantly decreased (*P* < 0.001) when the concentration of *Ts*Exos was above 100 μg/mL. When IPECs were incubated with *Ts*Exos for 3 or 6 h, the cell viability curve indicated a downwards trend with an increase in concentration, but for cells induced for 12 or 24 h, the cell proliferation curve showed an obvious downwards trend. In addition, at the 48 h time point, there was no effect on cell proliferation, suggesting that the exosomes had been degraded (Figure 2). Therefore, 150 µg/mL *Ts*Exos were selected as the working concentration of subsequent experiments and cocultured with cells for 12 or 24 h, reducing cell proliferation by 30%.

### Effects of *Ts*Exos on the physiological and biochemical states of IPECs

**Figure 3:**
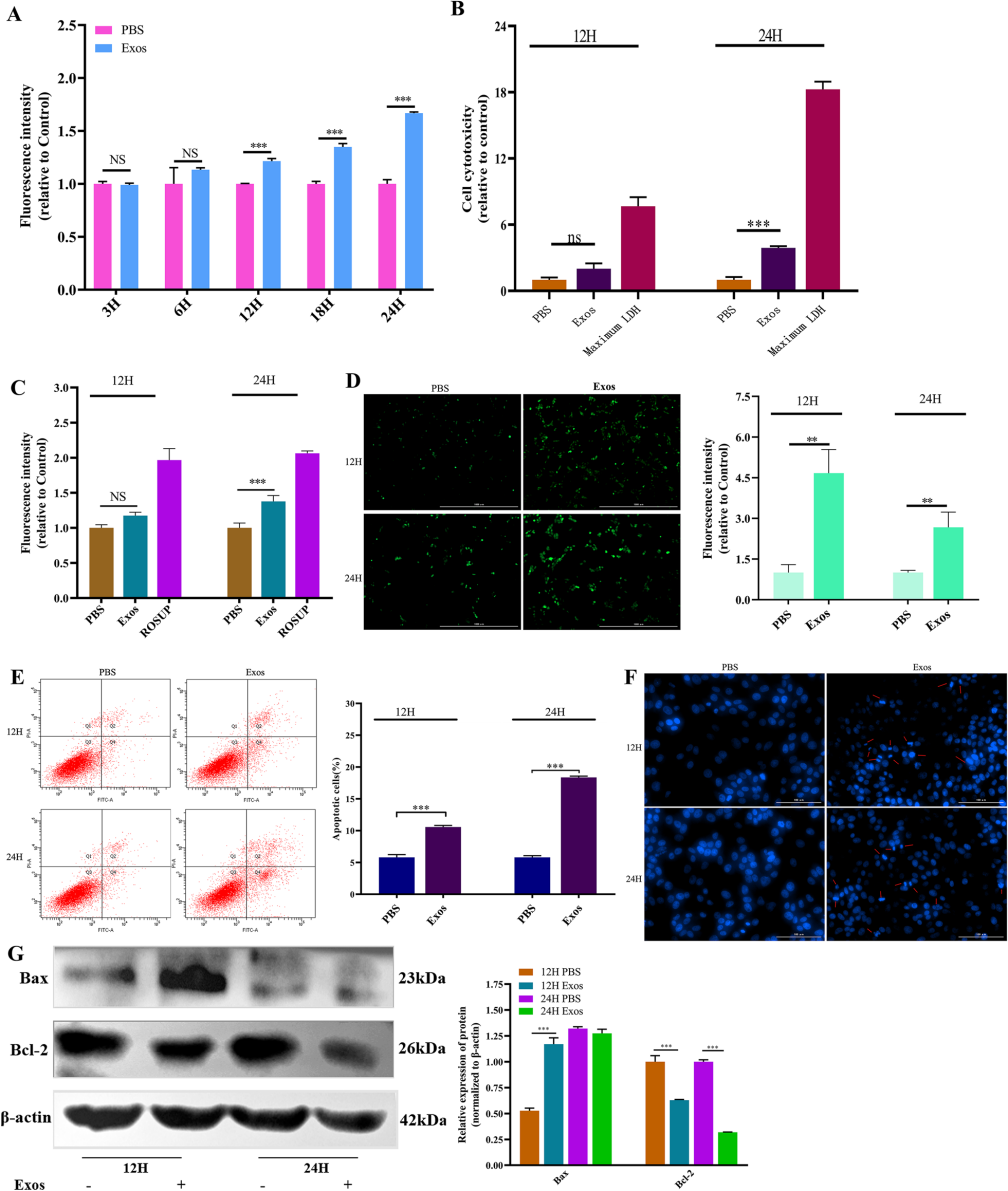
**Measurement of permeability, cytotoxicity, oxidative stress and apoptosis.** *Ts*Exos **(**150 μg/mL) were incubated with IPECs for different times, and then, the assays were carried out. **A** Cell permeability was determined by detecting the fluorescence intensity of FITC-dextran at 493-nm excitation and 517-nm emission wavelengths. **B** Cell cytotoxicity determined by assessing the level of LDH at OD_490_. Cellular oxidative stress was detected by a fluorescence microplate reader at 488-nm excitation and 525-nm emission wavelengths (**C**) and a fluorescence microscope with a magnification of 20× PL FL and a scale bar of 100 μm (**D**). Apoptosis of IPECs was determined by staining with annexin V and PI followed by flow cytometry (**E**), by staining with Hoechst 33258 (**F**) and by analysing the relative expression of Bax and Bcl-2 using Western blotting (**G**). All assays were performed in triplicate, and the data are presented as the mean ± SD. *P < 0.05, **P < 0.01, ***P < 0.001 compared with the IPEC + PBS group.

Based on the results of the *Ts*Exos on cell proliferation, experiments examining the effects of *Ts*Exos on cellular permeability, cytotoxicity, oxidative stress and apoptosis were carried out. First, to evaluate the cellular permeability of monolayer cells, we measured FITC-labelled dextran using a multifunctional microplate reader. The experiment revealed that after incubation of *Ts*Exos with IPECs for 12 h, the fluorescence intensity of FITC-dextran increased significantly compared with that of the control (*P* < 0.001), and the content of FITC-dextran continued to increase over time (Figure 3A). Moreover, we analysed the effect of *Ts*Exos on cytotoxicity by measuring the level of lactate dehydrogenase (LDH) at 490 nm. The results showed that there was no difference (*P* > 0.05) in the LDH content at the induction time of 12 h, but the level of LDH was obviously increased after coincubation for 24 h (Figure 3B). Subsequently, oxidative stress was estimated by measuring the level of ROS using a fluorescence microplate reader and a fluorescence microscope. As shown in Figures 3C and D, both methods suggested that the fluorescence intensity increased significantly at 24 h (*P* < 0.001; *P* < 0.01) compared with that of the control, while there was no difference (*P* > 0.05) between the cell treatment group and the cell control group at 12 h when the fluorescence intensity was evaluated by a microplate reader; however, a significant difference (*P* < 0.01) was observed in the evaluation by a fluorescence microscope.

Finally, the influence of *Ts*Exos on apoptosis was detected using FCM, Hoechst 33258 staining, and Western blotting. The results showed that the rates of early as well as late apoptosis induced by 12 or 24 h exposure to *Ts*Exos were 1.8 times or 3.2 times, respectively, compared with that of the control (Figure 3E). Furthermore, the results of cell apoptosis detected by Hoechst 33258 were consistent with the FCM results, which also verified that *Ts*Exos significantly induced the apoptosis of IPECs compared with the controls (Figure 3F). Western blotting was used to detect the expression of proapoptotic (Bax) and antiapoptotic (Bcl-2) proteins, and the grey values of each band were analysed by ImageJ software (Figure 3G). Similarly, the protein expression of Bax was significantly higher than that in the control at 12 h (*P* < 0.001), while the protein expression of Bcl-2 was significantly lower than that in the control (*P* < 0.001).

### Effects of *Ts*Exos on the innate immunity of IPECs

**Figure 4:**
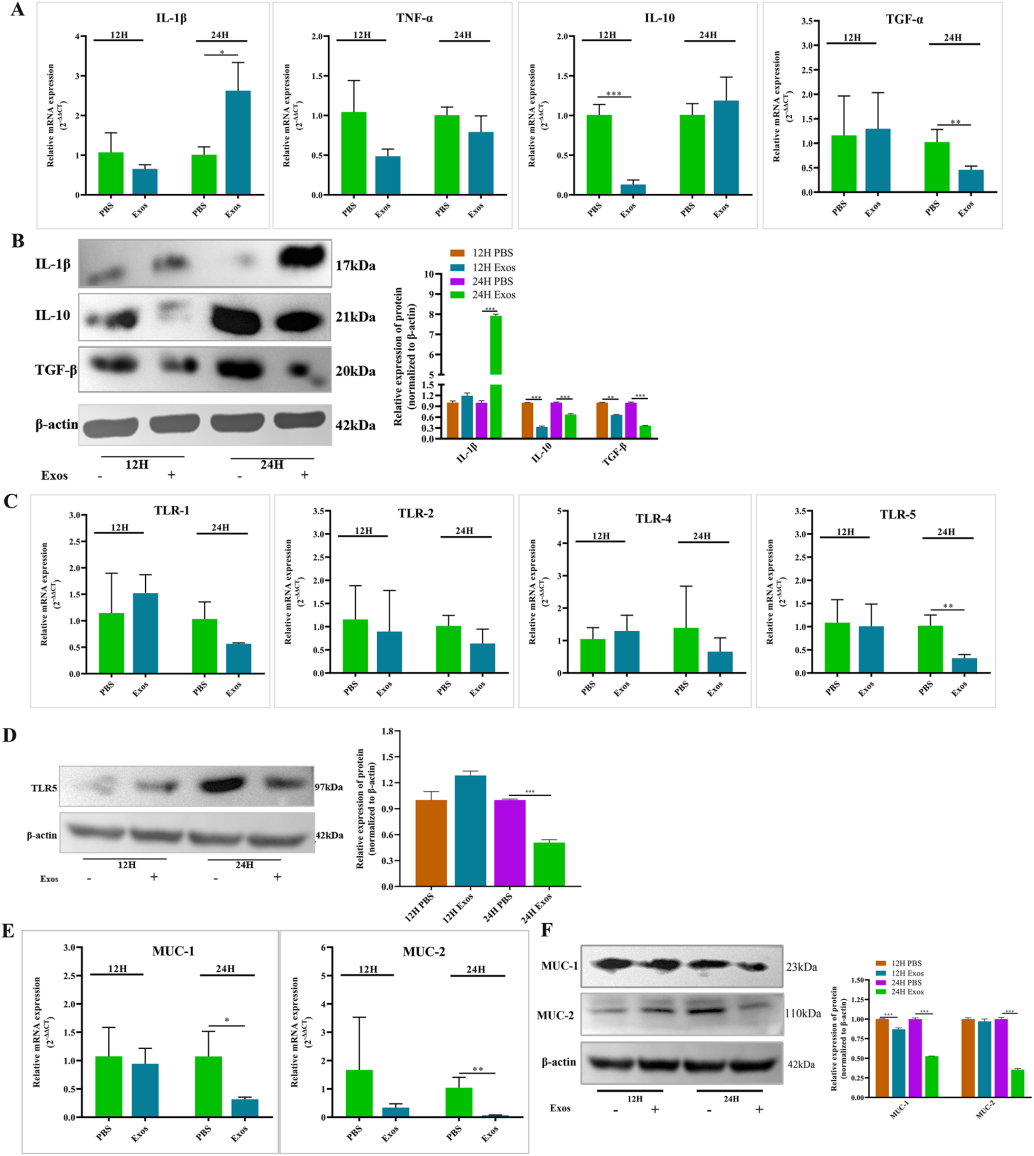
**Analysis of cytokines, Toll-like receptors and mucins in IPECs.** *Ts*Exos (150 μg/mL) were incubated with IPECs for different times, and then, the assays were carried out. **A** RT‒qPCR and **B** Western blotting were used to analyse the expression of cytokines produced by IPECs treated with *Ts*Exos. **C** RT‒qPCR and **D** Western blotting were used to analyse the expression of Toll-like receptors in IPECs incubated with *Ts*Exos. **E** RT‒qPCR and **F** Western blotting were used to analyse the expression of mucins in the IPECs cultured with *Ts*Exos. Assays were performed in triplicate, and the data are expressed as the mean ± SD. *P < 0.05, **P < 0.01, ***P < 0.001 compared with the IPEC + PBS group.

To analyse the effects of *Ts*Exos on the innate immunity of IPECs, we detected the transcriptional and protein levels of the cytokines, Toll-like receptors, and mucins produced by IPECs. RT‒qPCR was used to analyse the changes in the transcriptional levels of IL-1β, TNF-α, IL-10, and TGF-β, and the results showed that the production of IL-1β was significantly increased, while the cytokines IL-10 and TGF-β were both noticeably decreased (Figure 4A). To further confirm the changes in the cytokines produced by IPECs, we used Western blotting to detect the expression of IL-1β, IL-10, and TGF-β in different groups. The results of Western blotting were consistent with the results of RT‒qPCR (Figure 4B), which showed that IL-1β was upregulated and IL-10 and TGF-β were both downregulated after IPECs were induced by *Ts*Exos, demonstrating that *Ts*Exos could promote cellular inflammation.

Moreover, we used RT‒qPCR to detect the mRNA levels of Toll-like receptors (TLR1, TLR2, TLR4, TLR5) in IPECs. The results showed that only the transcriptional level of TLR5 was obviously higher than that of the control at 24 h (*P* < 0.01) (Figure 4C). The protein level of TLR5 was also found to be significantly decreased compared to that of the control at 24 h (*P* < 0.001) (Figure 4D).

We continued to detect the transcriptional levels of mucins (MUC1 and MUC2) in IPECs by RT‒qPCR. As Figure 4E shows, the mRNA levels of MUC1 and MUC2 were significantly decreased (*P* < 0.05; *P* < 0.01) relative to the controls after *Ts*Exos and IPECs were cocultured for 24 h (Figure 4E). Furthermore, the protein expression of MUC1 and MUC2 detected by Western blotting corresponded with the results of RT‒qPCR (Figure 4F), indicating that *Ts*Exos could induce low expression of mucin in IPECs.

### Effects of *Ts*Exos on the tight junctions of IPECs

**Figure 5:**
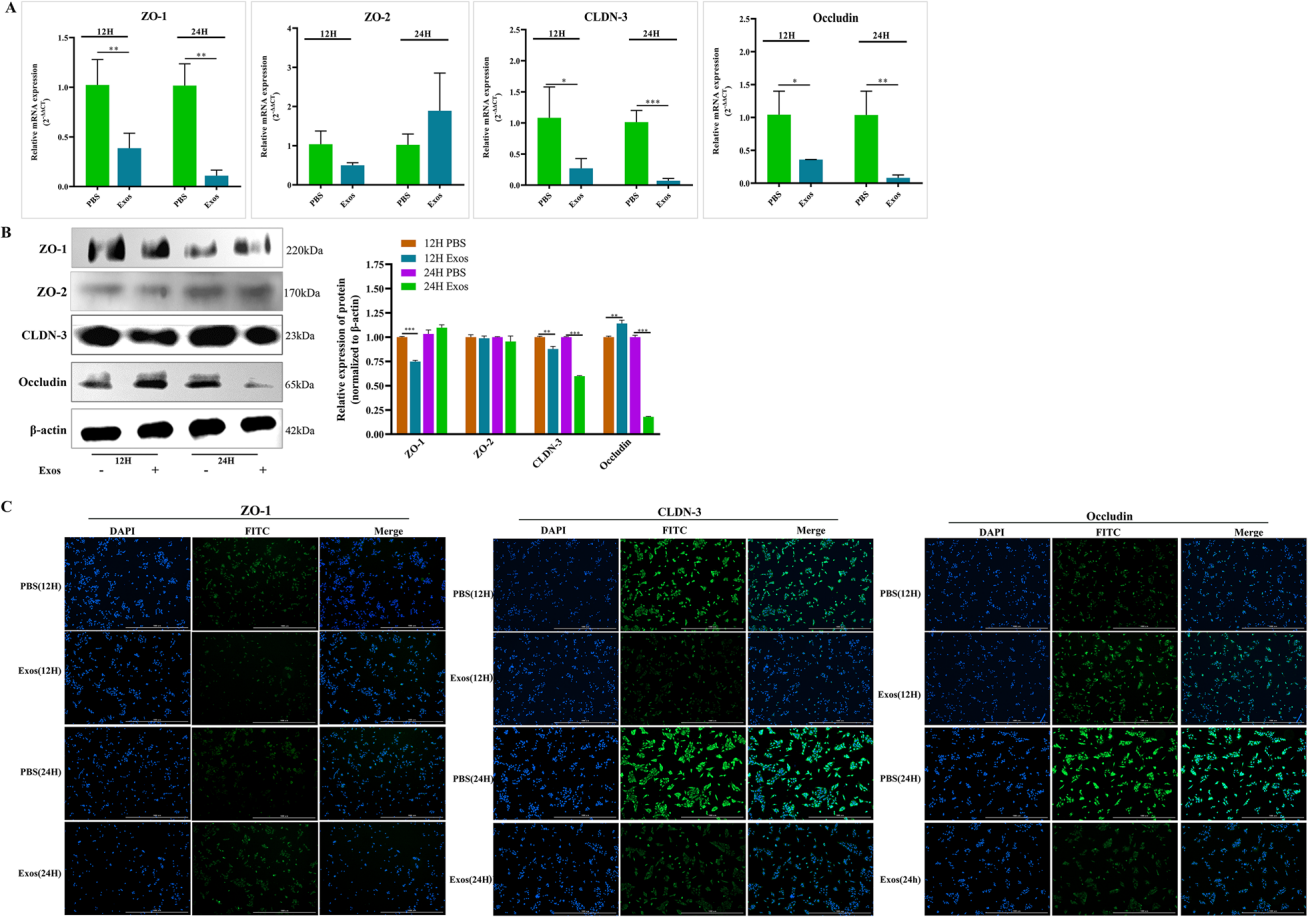
**Changes in tight junctions on IPECs.** *Ts*Exos (150 μg/mL) were incubated with IPECs for different times, and then, the assays were carried out. **A** RT‒qPCR, **B** Western blotting and **C** immunofluorescence with the magnification of 4× PL FL and the scale bar of 1000 μm were used to analyse the expression of cytokines produced by the IPECs treated with *Ts*Exos. Assays were performed three times, and the data are expressed as the mean ± SD. *P < 0.05, **P < 0.01, ***P < 0.001 compared with the IPEC + PBS group.

To evaluate the effects of *Ts*Exos on the tight junctions of IPECs, we determined the transcriptional and protein levels of tight junction-related proteins in IPECs. First, we used RT‒qPCR to analyse the changes in the transcriptional levels of different tight junction-related genes (ZO-1, ZO-2, CLDN-3, and Occludin). The results are shown in Figure 5A. *Ts*Exos significantly decreased the transcriptional levels of ZO-1, CLDN-3, and Occludin compared with the controls (*P* < 0.01, *P* < 0.05, *P* < 0.05) at 12 h, and the relative expression of mRNA was decreased much more at 24 h, while *Ts*Exos did not cause significant changes in the transcriptional level of ZO-2 (*P* > 0.05). Subsequently, we used Western blotting and immunofluorescence to further verify the RT‒qPCR results. The results of Western blotting showed that the expression levels of ZO-1 and CLDN-3 were significantly decreased at 12 h relative to those of the control (*P* < 0.001; *P* < 0.01), and *Ts*Exos led to low expression of CLDN-3 at 24 h. We also observed that the expression of Occludin was significantly upregulated compared with that of the control at 12 h (*P* < 0.01), which was in contrast to the results detected by RT‒qPCR; however, at 24 h, the expression level of Occludin was noticeably lower than that in the control (*P* < 0.001). No change in the protein expression of ZO-2 was revealed, which was consistent with the RT‒qPCR results (Figure 5B). Moreover, the immunofluorescence results showed the same trend as Western blotting, indicating that *Ts*Exos affected the tight junctions of IPECs by decreasing the expression of ZO-1, CLDN-3, and Occludin (Figure 5C).

## Discussion

Intestinal epithelial cells are the first line of defence against antigens, toxins, and harmful substances in the intestinal tract, acting both as a physical barrier and as a participant in intestinal mucosal immunity [21]. Therefore, the balance between cell viability and cytotoxicity, injury, apoptosis, and the inflammatory response is the key to maintaining the normal barrier function of epithelial cells [22]. After muscle larvae of *T. spiralis* are ingested by the host, the worm is released into the intestinal tract, and it completes its life cycle phases, such as invasion, colonization, and breeding. Studies have recently shown that exosomes derived from various parasites play an important role in information exchange, interaction with host cells, and avoidance of immune rejection [23–26]. Therefore, exploring the mechanism of the exosomes secreted from *T. spiralis* in the process of its invasion is important for the prevention and control of trichinosis.

In this study, IPEC-J2 cells were treated with different concentrations of *Ts*Exos for different durations, and the results showed that cell proliferation was decreased following the increase in concentration and time, but there was no difference in cell viability at 48 h. Finally, we selected 150 µg/mL *Ts*Exos as the working concentration for subsequent experiments and cocultured *Ts*Exos with cells for 12 or 24 h, reducing the rate of cell proliferation by 30%. Liang et al. [27] showed that the viability of Levo cells was significantly reduced after 48 h of stimulation with 100, 150, and 200 μg/mL exosomes derived from *T. asiatica* adult worms, which was consistent with the concentration of *Ts*Exos in our study, but the induction time was different.

Studies have found that extracellular vesicles from the parasite *Fasciola gigantica* are associated with increased levels of reactive oxygen species in human intrahepatic biliary epithelial cells and induce autophagy and DNA damage and repair processes [28]. Lisette et al. [29] showed that the incubation of cells with extracellular vesicles of trypomastigotes of the Pan4 strain of *T. cruz*i induces a number of changes in the host cells that include a change in cell permeability and higher intracellular levels of Ca^2+^. Therefore, we conducted a series of experiments to study whether *Ts*Exos also influenced the physiological and biochemical processes of intestinal epithelial cells. Based on the effects of *Ts*Exos on proliferation, this study discussed the effect of exosomes on the barrier function of intestinal epithelial cells, including permeability, cytotoxicity, oxidative stress and apoptosis. The results showed that *Ts*Exos ingested by IPECs could lead to a series of changes, such as increased permeability, increased levels of cytotoxicity and oxidative stress, abnormal apoptosis, and regulation of the cellular response.

The tight junctions of epithelial cells are the basis of maintaining barrier function and a normal physiological state, and the interruption of tight junctions can significantly change the permeability of cells, allowing pathogens to easily invade the organism, which is a sign of many pathological states. With further studies, more cytokines (inflammatory factors, chemokines, tumour necrosis factors) have been proven to affect tight junctions in various in vivo and in vitro studies and have been shown to regulate the body’s homeostasis and stress response [30, 31]. In addition, early studies have shown that different TLRs participate in the regulation of tight junctions during pathogenic infection and play an important role in maintaining the integrity of the intestinal epithelial barrier [32]. The proportion of mucin in the complete intestinal mucosal barrier remains balanced. Once the intestine is infected by pathogens, it will affect the expression and distribution of mucin [33]. Therefore, we further studied the effect of *Ts*Exos on the innate immunity of IPECs. This study found that *Ts*Exos could induce the upregulation of IL-1 and the downregulation of IL-10 and TGF-α in IPEC-J2 cells to promote inflammation. The expression of TLR5 decreased significantly, suggesting that *Ts*Exos regulate the barrier function of epithelial cells by affecting the expression of TLR5. In this experiment, the expression levels of MUC-1 and MUC-2 were lower, which might be related to the survival of the parasite under the attacks on the host.

The tight junctions of cells are the key to maintaining the function and integrity of the epithelial barrier, which mainly depends on tight junction proteins [34]. In this study, IPEC-J2 cells were induced by *Ts*Exos, and the results showed that the expression levels of ZO-1, CLDN-3, and Occludin were significantly downregulated, indicating that *Ts*Exos affected the tight junctions of IPECs. A study (2021) showed that the serine protease in the ES antigen of *T. spiralis* reduced the expression of tight junction proteins in Caco2 cells through the MAPK signalling pathway [35], which was similar to the effect of *Ts*Exos on tight junction proteins in our study.

In conclusion, *Ts*Exos participate in the regulation of various life activities of cells after entering IPEC-J2 cells. *Ts*Exos can reduce cell viability, improve permeability, cause cell damage, cause excessive oxidative stress, and cause abnormal apoptosis. Furthermore, *Ts*Exos promote the inflammatory response of cells, affect the signal transduction mediated by Toll-like receptors, and destroy the cellular mucin defence system and the tight junction of cells. Therefore, exosomes play an important role in the process of *T. spiralis* invasion in the host.

## Data Availability

The datasets used or analysed during the current study are available from the corresponding author on reasonable request.
